# Ben Crewe on the Bench? Bringing the Dimensional Pains of Punishment into the Courtroom

**DOI:** 10.1177/0306624X231159885

**Published:** 2023-03-18

**Authors:** David Hayes

**Affiliations:** 1 The University of Sheffield, Sheffield, South Yorkshire, UK

**Keywords:** sentencing, subjective experience, pains of punishment, proportionality, personal mitigation

## Abstract

Penal subjectivists argue that the severity of punishment ought to be measured in terms of penal subjects’ actual experiences, rather than that intended by sentencing authorities. One challenge that subjectivists must confront, however, is that it is difficult to meaningfully compare the subjective experiences of different individuals, in a way that is sufficiently equitable and consistent to satisfy the requirements of just sentencing. This paper considers the prospects and pitfalls of Ben Crewe’s *dimensional* approach to the pains of imprisonment as a means of overcoming this challenge during sentencing. Crewe’s ground-breaking work takes the “deprivations and frustrations” of everyday prison life associated with Gresham Sykes, and subjects them to four spatial metaphors that help to trace differences between penal experiences: depth; weight; tightness; and breadth. The applicability of this approach to sentencing decision-making is considered, and implications are drawn for sentencing research agendas.

## Introduction

From its humble beginnings in the sociology of a New Jersey prison (Sykes, 1958), the study of the “pains of punishment” has become a major component of the descriptive sociology of punishment. Although still well-studied in prisons (e.g., Crewe, 2011), pain has proven to be a highly adaptable and mobile concept, applicable to a range of non-custodial penalties (e.g., Durnescu, 2011; Hayes, 2015; Payne & Gainey, 1998; and compare Nugent & Schinkel, 2016), to radically different sorts of imprisonment (e.g., de Vos & Gilbert, 2017; Sexton, 2015; Shammas, 2014), and indeed, beyond the formal limits of the penal system (e.g., Gashi et al., 2021; Harkin, 2015; Skinns & Wooff, 2021).

But despite this level of interest, research into pain (and other subjective experiences of punishment) has had relatively little purchase in more normative debates about sentencing practice. Although there was some debate about 10 years ago about the extent to which subjective experiences should be treated as a part of punishment (Bronsteen et al., 2009, 2010; Gray, 2010; Kolber, 2009a, 2009b; Markel & Flanders, 2010; Markel et al., 2011; see also Ryberg, 2010), there has been surprisingly little interest in the relationship between penal philosophy, sentencing practice, and the subjective experiences of punishment. Indeed, most contemporary references to the debate between penal objectivists (who argue that punishment consists in what is intended to be punishment) and subjectivists (who argue that punishment consists in what is actually experienced by the subject) tend to be cursory and dismissive (e.g., Haque, 2013, pp. 79–80; von Hirsch, 2017, pp. 68–69). Despite an ongoing trickle of empirical scholars with an interest in the relationship between subjective experiences and penal theory (e.g., Hayes, 2016; Khechumyan, 2018; Schinkel, 2014b), the debate seems largely to have subsided, the objectivist orthodoxy having weathered the subjectivist storm.

The fact that penal theorists have largely turned their backs on the possible relevance of penal subjects’ actual experiences is surprising in the context of the surfeit of pain analyses, precisely because part of the subjectivist/objectivist debate centered on how much we can actually know about pain. Pain is, after all, amorphous, intimate, and incomparable: what is my unendurable torment could be your mere nuisance (Christie, 1981). For objectivists, the sheer complexity of pain means that it cannot be fairly or consistently measured, and that it would therefore be impossible to apportion levels of pain that were fair, just, and proportionate to the severity of an offense. Given that a judge cannot predict all the circumstances of a penal subject’s life at the point of sentence, how are they to ensure that offenses of similar seriousness and treated sufficiently similarly? For this reason, punishment is normally defined and measured in terms of what would be “normally considered unpleasant,” rather than in terms of what is actually experienced (e.g., McPherson, 1967).

However, as I have argued elsewhere (Hayes, 2018), that objection relies upon an unduly pessimistic view of the capacity of the social sciences to predict the pains of punishment likely to arise in a particular case. Given the proliferation of sociological studies of the pains of punishment, there is a wealth of potential information that could be taken into account at the sentencing stage. The purpose of this paper is to explore how we might do so, as a means of highlighting further research agendas, encouraging greater collaboration between empirical and normative researchers in this field, and reinvigorating the argument for penal subjectivism. It will focus, in particular, on the *dimensional* model pioneered by the English prison sociologist, Ben Crewe (with colleagues and alone). Crewe deploys a series of metaphors (depth, weight, tightness, and breadth) that map punishment in spatial terms. These spatial dimensions suggest a way of comparing different pains of punishment against one another, in exactly the way that penal subjectivists argue that sentencing authorities should. This paper therefore explores the extent to which Crewe’s approach could be used in a sentencing context to take greater account of differences in subjective experiences of punishment. Although Crewe’s framework is well-developed, and while its spatial dimensions seem intuitively well-suited to making the sorts of comparisons that sentencers must, I use it here only as an example, to demonstrate the ability of sentencing to absorb this sort of empirical data, and the specific requirements that sentencing imposes on such data. Crewe’s account is by no means the only or best framework for this purpose; it is used only to explore the issues that (more) subjectivist sentencing would raise.

This paper begins with a brief outline of Crewe’s account of the dimensional pains of imprisonment, before considering the essential features, strengths, and drawbacks of a model of sentencing based upon that account. It concludes that, while Crewe’s account cannot be straightforwardly applied to a sentencing context, it does illustrate some of the gains that could be made, suggesting that methodological rejections of penal subjectivism are at least overstated. This should encourage further discussion of penal-subjectivist approaches to measuring penal severity (including for sentencing purposes), and more detailed attempts to make the subjective experiences of punishment practically available to sentencing authorities as a matter of policy and practice. The fact that we *could* take greater account of the pains of punishment in sentencing, in other words, should reinvigorate debates about whether and in what ways we *should*.

Before any of this, however, a quick caveat. While the pains of punishment, including Crewe’s own framework (e.g., Ystanes & Ugelvik, 2020), are amenable to application to different jurisdictions around the world, sentencing is inherently bound by the precise processes, practices and institutions of the jurisdiction in question. I will speak throughout in terms of English sentencing, since England and Wales is my home jurisdiction and the sentencing regime with which I am most familiar. By placing things in an explicitly English context, I wish to draw attention to my own perspective and the situated assumptions that underlie my account, not just in terms of institutions and practices but also in terms of values and expectations about what goes into sentencing. The aim is to draw out precisely that which is specific to England and Wales, so that readers from other jurisdictions can account for them when considering the implications of my argument for their own criminal justice systems.

## The Pains of Imprisonment: Crewe’s Dimensional Metaphors

The concept of the “pains of imprisonment” was originally rather amorphous, consisting of any deprivation or frustration that represents a fundamental challenge to the penal subject’s wellbeing or sense of self (Sykes, 1958, p. 64). This served the purpose of Sykes’s (1958) wide-ranging analysis, which was after all concerned with the wider everyday life of his subject prison. But this shapelessness is limiting from a sentencing perspective. On Sykes’s account, we could say that a particular pain was present or absent, but not much more than that. Indeed, since pain is necessarily a subjective and personal experience (e.g., Christie, 1981, pp. 9–11), the more the pains of imprisonment had to describe multiple subjects’ experiences, the less detail they could meaningfully convey.

These are fundamental challenges facing any account of the pains of punishment today—pain is such a nebulous and personal concept that we cannot approach it objectively, only *inter-subjectively* (see generally Hayes, 2016). However, Crewe’s (2011, 2015) account does take us further than Sykes’s (1958) in that it imposes a *dimensionality* onto the punishment, developing and adding to metaphors originally identified by Downes (1988) and King and McDermott (1995). Generally speaking, we only measure sentences in terms of their *length*—the period of time for which the sentence must be endured (compare Kolber, 2009a). To be sure, the *mode of punishment* deployed also affects sentence severity: prison, for instance, is generally considered harsher than probation. But the only factor that distinguishes between two sentences involving the same mode of punishment is length. However, as a matter of descriptive reality, prisons (and other sites of punishment) can differ enormously in terms of their architecture, population, regimes, and culture. It is never possible to say that three years in one prison is much the same as three years in another, making length an imperfect analogy for the actual harshness of a sanction. Crewe’s research is therefore helpful, in that it supplements length with four other axes on which punishment can become more or less painful: *depth*, *weight*, *tightness*, and *breadth* (see generally Crewe, 2011; Crewe et al., 2014; Crewe, 2015, 2021; Crewe & Ievins, 2021).

### Depth

The *depth* of punishment, Crewe’s first dimension, is a question of how totally the penal regime imposed upon the subject consumes them (Crewe, 2011; Downes, 1988, p. 521). We tend to think of prisons as “total” institutions (Goffman, 1961), which isolate and separate the offender from the non-penal world. This is oversimplistic, especially in an era of prison visitation, telecommunications, and (limited) internet access. No (modern) prison has ever been a mere oubliette. But certainly, some prison regimes are more total than others: a prisoner subjected to solitary confinement in an American supermax facility is more deeply “swallowed” up than someone in a Swedish open prison. Moreover, we can say that, *in general*, custodial sentences impose higher barriers between the penal subject and the non-penal world than a non-custodial sentence would.

It does not follow that a “deeper” penalty will involve more subjective pains, all else being equal. After all, it can be painful to interact with the wider community in the context of a punishment, and prison can insulate the subject from that (e.g., Hayes, 2018; Nugent & Schinkel, 2016). Indeed, Crewe (2021, pp. 341–345) notes that some more open prison orders can actually feel deeper for prisoners, in that they expose the prisoner to the outside world from which they have been removed, making the distance feel much greater, subjectively (cf. de Vos & Gilbert, 2017). Despite this complexity, depth *is* associated with particular pains—notably, those associated with detachment from support networks, loss of opportunity, and uncertainty about the future (Crewe, 2011).

### Weight

Crewe’s second dimension, *weight*, concerns the psychological onerousness and oppressiveness of a punishment: the extent to which it bears down upon its subject (Crewe, 2011; King & McDermott, 1995, p. 521). An experience will be *heavy* if it imposes on the everyday life of the penal subject, by imposing rules and regulations on every aspect of the subject’s life, or by invading their thought processes in an onerous way. Thus, a supermax prison might be heavy and an open prison light in terms of the prison regime. However, weight can also be associated with rehabilitative treatment programs that force a confrontation with the self or expect change, helping to explain some of the bite of the “pains of rehabilitation” (Hayes, 2015, pp. 91–93; compare Nugent & Schinkel, 2016). Not only can these pains contribute to “tightness,” in the sense that one is subject to oversight, but they can also force a confrontation with one’s past identity, or require difficult, even painful lifestyle changes.

Interestingly, however, Crewe et al. (2014) offer an important modification to this concept of weight as invasive presence. They observe that prisoners can feel pain in a light regime that is neglectful or scornful, and may find support or even comfort in a regime that is a constant presence in their lives, providing order, stability, and safety to which they might not be used (cf. Liebling, 2004). As a result, Crewe et al. (2014) propose a second nested dimension within weight: there is an opposition not just between *heavy* and *light*, but also between the *presence* or *absence* of a regime, creating four possible combinations (see generally Crewe et al., 2014, p. 397). Pains may be *heavy-present* if they are oppressive and intrusive, but also authoritative, visible, and interventionist—such as a paternalistic form of regulation “for your own good.” By contrast, the example that Crewe et al. (2014, p. 404) offer of a *heavy-absent* regime is of a prison with a restrictive regime, but where the prison staff have retreated from landings. The prison regime is strict and oppressive, but offers none of the protection that might be expected to come with that.

Crewe et al.’s (2014) central argument is that “lightness” does not necessarily and automatically create a less painful environment. Indeed, a *light-absent* regime, which is characterized by relative openness and cooperation between penal staff and subjects, but also by a lack of authority and by the invisibility of power relations, can be very difficult to endure, due to a lack of consistency, clarity, and safety within the penal regime. The “holy grail” of prison legitimacy (Crewe et al., 2014, p. 404) is the *light-present* regime, which is not overly oppressive while still relating to and investing in prisoners, creating a safe and secure community without imposing order unfairly or exerting undue oppressive control.

### Tightness

Crewe’s third dimension, *tightness*, is a reflection of changing prison regimes within England and Wales over the past several decades. Crewe (2011) notes a tendency for prisons to exert control over the individual less by locking them in a deep hole, or weighing them down with direct oppressive force, but rather with increasingly *tight* regimes of surveillance and review. Tightness is particularly associated with a cluster of pains, including those of: the uncertainty and indeterminacy of punishment; omnipresent psychological assessment; and the regime’s expectations of self-government (Crewe & Ievins, 2021, pp. 49–50). Tightness in English prisons, then, allows technical freedom (subject to the restrictions of the regime), but with the knowledge that how one uses that freedom will be subject to surveillance, cataloging, analysis, and review that will factor into decisions about, for example, early release (cf. de Vos & Gilbert, 2017; Shammas, 2014).

However, although tight regimes may sound panoptic, or even Orwellian, they are not necessarily experienced subjectively as more painful than “loose” ones. Crewe and Ievins (2021, pp. 53–58) note that a regime can feel *simultaneously* loose and tight—for instance, if penal surveillance leads to opaque decision-making that is experienced as inconsistent, neglectful, or even random. Penal subjects might *expect* to be gripped tightly by a (rehabilitative) penal regime, and experience looseness as neglect or negligence in the exercise of the State’s power to manage risk (Crewe and Ievins, 2021, pp. 60–64). Indeed, insofar as a “tight” regime attempts to change penal subjects’ modes of thinking, a “tight” regime may not be experienced as painful at all, so much as a realization of one’s new pro-social identity and attitudes. In this sense, tightness is easier to endure, even desirable, when it is combined with *recognition*—when it is perceived as a legitimate or supportive engagement with the subject. By contrast, looseness can feel like *misrecognition*—as being seen badly, or not really seen at all (cf. McNeill, 2019). Once again, therefore, tightness needs to be understood as two-dimensional in its own right: on one axis, a penalty may be tighter or looser; on the other, that “grip” may be supportive and responsive, or top-down and neglectful. Generally speaking, a tight and neglectful regime will be more painful than a tightly supportive one, and we would expect a loose and supportive one to be less painful still. However, these conclusions are difficult to test empirically, and are intimately related to the subject’s own experience of, and attitudes to, the penal State’s attempt to mold them.

### Breadth

Crewe’s final dimension, breadth, describes the extent to which punishment saturates all aspects of the penal subject’s life. Whereas depth is the extent to which punishment plucks one out of one’s wider social context, and holds one apart from sources of pleasure and support, breadth is the opposite: the “penetration of penal control into civil society” (Downes, 1988, p. 187). While the prison might therefore be thought of as “narrow,” it has various emanations that increase its breadth, from the oversight associated with release on license, to stigmata that physically or psychologically mark prisoners after release—such as an inability to cope with the comparatively unregimented nature of “outside” life (Crewe, 2015, p. 60).

The introduction of the “breadth” dimension substantially improves Crewe’s framework’s ability to engage with a wider range of punishments. It can capture pains that are relatively unique to non-custodial penalties, which are characterized by their taking place alongside existing social responsibilities and connections. Such pains include the difficulties of navigating punishment as an extra obligation alongside matters as diverse as employment, childcare and responsibility for other dependents, seeking welfare or other benefits, and physical or mental healthcare—some or all of which the penal subject must manage for themselves (see generally Hayes, 2015; Nugent & Schinkel, 2016; van Ginneken & Hayes, 2017). Thinking in terms of the breadth of punishment—of how deeply it extends into everyday life—can help us to account for these phenomena, and properly decide how and to what extent to attribute them to a punishment (compare Hayes, 2018).

## Applying Crewe’s Metaphors in a Sentencing Context

Having sketched Crewe’s dimensional model, we can now consider how to use it in a sentencing context. I will begin by situating this account in the English sentencing model for the non-English reader, before applying Crewe’s dimensions to that system. This will enable us to explore the potential strengths and drawbacks that Crewe’s account might bring to the sentencing stage.

### Situating the Perspective: The Anglo-Welsh Sentencing Model

Sentencing in England and Wales is governed by judicial discretion, but this discretion is increasingly closely constrained by sentencing guidelines, put out by the Sentencing Council. Judges must generally abide by these guidelines, unless doing so would be contrary to the “interests of justice” (Coroners and Justice Act 2009, s. 125). Each guideline contains a series of factors to be taken into account, but for present purposes, we may divide these into three core phases: the initial determination of offense seriousness; the weighing up of aggravating and mitigating factors; and the consideration of additional statutory considerations.

In the first phase, the sentencer is required to consider two components of offense seriousness: the *harm* that the offense has caused, and the penal subject’s *culpability* for it. Individual guidelines provide an exhaustive list of factors to take into account when considering the offense. Weighing up these factors, the sentencer must then rate each of the two components as either higher or lower. An offense that is rated as high harm/high culpability will be a “Category A” offense. A “Category B” offense is ranked “high” in terms of one component and “low” in the other, and a “Category C” offense is ranked “low” in terms of both. These categories then specify a limited range in which sentences may be set (say, between 6- and 30-months’ imprisonment), and a starting point in the middle of that range (in our example, 18 months’ imprisonment) that informs the second phase.

Once the starting point and available sentencing range are determined, sentencers are presented with a list of aggravating and mitigating factors to consider. In this stage, the list is *non-exhaustive*, enabling sentencers to respond to the specific circumstances of individual sentences, within the limited range of discretion provided in the first step. Aggravating and mitigating factors may relate to the offense, the victim, and wider circumstances of the case, but may also touch upon *personal mitigation*—that is, characteristics of the offender that are relevant to their likely experience of the punishment (e.g., Roberts, 2011). Using aggravating and mitigating factors, judges must weigh up where in the available range the sentence should fit, starting from the specified starting point.

The third stage, which is listed in guidelines as a number of separate steps, consists of specific considerations that are imposed upon the sentencer by statute. These may be general considerations, applicable to all offenses (such as the reduction of sentence for an early guilty plea), or they may be specific to types of offenses (for instance, there may be a mandatory minimum sentence for repeat offenses). Each of these stages is applied after determining the “proper” (proportionate) sentence in the first two steps, and therefore constitute a modification of it, rather than part of that process.

English sentencing guidelines therefore impose a strict concern with proportionality on the sentencing judge, whose discretion is constrained by the level of offense seriousness (unless there are exceptional “interests of justice” reasons to go beyond those constraints). This is matched in the general structure of sentencing law, which limits imprisonment and community penalties behind offense-seriousness thresholds. That is, a sentencer cannot impose a community order unless the offense is serious enough that another non-custodial penalty would not be proportionate to the offense (Criminal Justice Act 2003, s. 148(1)). Likewise, a prison sentence (whether suspended or immediate) cannot be imposed unless the offense is too serious for either or both of a community order or a fine to be proportionate (Criminal Justice Act 2003, s. 152(2)).

### Using Crewe’s Dimensional Pains of Punishment in the Courtroom

Logically, there are two places where Crewe’s model could be brought into the process outlined above: at the point of identifying sentencing ranges; and at the point of identifying aggravating and mitigating factors. In the first instance, we could bring Crewe’s dimensions into play when identifying the appropriate maximum and minimum sentences, and the starting point sentence for an offense involving a given level of harm and culpability. Instead of making ranges a matter solely of the length of a sentence, we could take account of the combined effect of the length, depth, weight, tightness, and breadth associated with a sentence to form the upper and lower limits of a sentence. This would require the quantification of the qualitative pains that Crewe identifies—for instance, each dimension could be assigned a score from “1” to “10,” and from the interaction of those scores, an appropriate length of sentence could be suggested. To be sure, this would be arbitrary (as I discuss further below). However, it would be less arbitrary, to the subjectivist, than an account based only on sentence length.

The second alternative is to take account of the dimensions of a punishment through the recognition of likely depth, tightness, weight, and breadth as mitigating factors, when deciding which precise length of what sentence to impose. For instance, a judge might recognize that a given prison is known to be particularly deep, and therefore mitigate the sentence accordingly.^1^ This approach would require much less amendment of existing procedures, and would probably be easier for judges to work with in practice, since it keeps the familiar output of a certain length of sentence. It would, however, come at the cost of making Crewe’s dimensions subservient to the length of the sentence: length is still the primary consideration, and sentences can only be mitigated if they are likely to prove particularly painful along one of Crewe’s axes. It is likely that patterns associated with existing mitigating factors would be repeated here. For instance, there is some evidence that English judges tend to find it easier to use aggravating factors to turn a harsh community penalty into a prison sentence than they do to mitigate a short prison sentence into a community penalty (Padfield, 2011). If the experience of punishment is relegated to the status of a mitigating factor, then it is unlikely that they will have a significant effect on this trend.

In short, each approach carries advantages and disadvantages from the sentencer’s perspective, and there is no reason to choose definitively between them here. My only purpose in this paper is to demonstrate that subjective experiences *can* be considered more thoroughly in sentencing; not to prescribe a specific set of practices and procedures. Indeed, whether as part of the overall calculation of severity in terms of “pain units,” or as a series of mitigating factors, Crewe’s taxonomy is attractive from a penal subjectivist approach to sentencing in three respects, to which the next three subsections turn. Firstly, Crewe’s taxonomy provides a valuable series of metaphors for *mapping* subjective experiences in the courtroom. Secondly, it helps to address some practical problems a purely *duration*-based metric of sentencing severity. Thirdly, it makes it easier to parse through questions of *commensurability* between different modes of punishment. In each case, we will discuss these advantageous features, and consider any pitfalls and limitations arising from each of them, before providing an overall discussion of the suitability of Crewe’s taxonomy as a guide to sentencing.

### Mapping the Pains of Punishment at Sentencing: Crewe the Cartographer?

The first, intuitive appeal of Crewe’s account is that, by measuring the pains of punishment in dimensional terms, it seems to enable us to *map* the pains of punishment onto a pseudo-spatial grid that enables direct comparisons to be made between different sentencing options. Crewe’s dimensions provide ways of speaking about how the treatment and governance of penal subjects (especially prisoners) *impact upon* their overall experiences of pain as a result of their punishment. The multitudinous and diverse pains of punishment are translated into a series of phenomena, whose influence we can begin to chart out through robust penological enquiry. Crewe’s taxonomy focuses and filters the ever-changing muddle of pains actually experienced by the subject through the lens of what is done to them, reducing the diversity of pains in a way that makes it easier to distinguish them from other, background pains of everyday life that are not related to the punishment (cf. Hayes, 2018).

This initial sense of cartographical certainty is, however, undercut by the fundamentally binary and qualitative nature of Crewe’s oppositions, which lack the capacity to allow fine distinctions between cases. For instance, we know from Crewe (2011) that punishments may be deep or shallow, tight or loose. We may be able to say that a given experience is heavier for some or all offenders than others, but we could not say that it was “3 kg heavier”—that would be to stretch the metaphor of “weight” to breaking point. However, length does offer such a quantum: we can say not just *that* a sentence is longer or shorter than another, but also *precisely how much* longer. The problem is that a sentence, especially a sentence seeking to be proportionate, parsimonious, or otherwise limited in its impact, *needs* the precise quantum that length, however imperfectly and arbitrarily, offers.

This means that any attempt to genuinely map Crewe’s binary oppositions to the degree needed for sentencing would require some sort of quantification of the qualities of penal suffering. Any such quantification would be arbitrary in two unavoidable ways. Firstly, no one person’s pain is like another’s—and we have no means of experiencing the pain of another for ourselves, locked as we are within the sensory limits of our own bodies (Christie, 1981). Pain is complex, intimate, and unique to the individual, and any attempt to boil it down to a 10-point scale will be arbitrary. It will inevitably fail to capture nuances in how pain affects individuals in different circumstances, at different times, and in different places. Secondly, any attempt to quantify pain *at sentencing* will then be additionally arbitrary in that the sentencer is attempting to *predict* the likely experiences that a sentence will cause. Any prediction, outside of the realm of science fiction, is vulnerable to error, which may well lead to an overly severe (or indeed, overly lenient) sentence in practice when unexpected, unrecognized, or misunderstood factors affect the actual experienced sentence.

Both the compression of information inherent in quantification, and the uncertainty of pain predictions are major problems from the normative stance of a penal subjectivist, for whom recognition of the experienced nature of punishment is crucial if we are to measure penal severity effectively. However, as I have already discussed elsewhere (Hayes, 2018, 2019, pp. 177–182), these are also both charges that subjectivists level against objective measurements of penal severity. The problem with measuring punishment in terms of length and type of sentence alone is precisely that it *ignores* the differences arising from the subjective experience of punishment, glossing over qualitative details and preventing any attempt at predicting subjective outcomes. Without wishing to downplay the arbitrariness of such a quantitative scaling of Crewe’s dimensions of punishment, in other words, it would at least *improve* courts’ abilities to account for subjective factors that vary the experience of sentencing. A bad prediction, after all, is more of an attempt to model the subjective experience of punishment than no prediction at all. This is especially so as, with further mixed-methods research, the arbitrariness of any quantitative scale could be reduced over time.

### Beyond Duration: Crewe the Complicator?

The second major advantage is that any use of Crewe’s taxonomy in sentencing decision-making would cause a cognitive decentring of the length of a sentence as the only axis of its severity, in the minds of both sentencers and the general public. The penal-subjectivist critique of existing sentencing practice is partly that it treats duration as possessing or representing aspects of penal severity that it does not or cannot capture (Kolber, 2009a, 2009b). However, if we take account of not just the length of a sentence, but also its other dimensions, we are at least closer to those differences in experience and in impact that can frustrate efforts to treat like cases alike (Ryberg, 2010). This allows much more nuanced consideration of the relative severity of custodial and non-custodial sentences.

The best way to challenge the primacy of duration in discussions of penal severity would be to integrate Crewe’s four dimensions at the stage of determining the starting range of sentence. If a sentencer must choose from a range not between six- and 18-months’ imprisonment (say), but between 25 and 45 “pain units,” then length is demoted to only one consideration amongst five. By contrast, if Crewe’s dimensions were only introduced later, as mitigating factors in the second stage laid out above, then duration is still the primary determinant—the penal language or quantum into which the other four factors are translated. However, even here, the length of a sentence could be much more responsive to its expected breadth, depth, weight, and tightness than is currently the case. Once again, it is more detail than sentencers currently have. Although partial and arbitrary, sentencing would be *less* partial and arbitrary, which can only be to the good.

That being said, either attempt to use Crewe’s dimensions runs into the challenge of working through how they *interact*. Simply put, Crewe’s scholarship tells us that there are certain tendencies in prison regimes. However, it is not possible, on the basis of Crewe’s concepts, respond to three different aspects of the process of comparison that are intrinsic to sentencing. Firstly, on Crewe’s account, one cannot say that depth and tightness both have the same potential impact upon the experienced severity of a punishment. Is the tightest regime as likely to cause subjective suffering as the deepest regime, or is the relative depth or shallowness of a punishment capable of affecting the overall severity more? Crewe’s analysis cannot really answer this question. It provides no means of comparing the relative intensity of different dimensions of punishment relative to one another.

Secondly, as we saw above, the dimensions of depth, weight and tightness were not always perceived by offenders as one-dimensionally painful. A heavier sentence might be less difficult to endure if it was the right kind of heaviness (Crewe et al., 2014), while a tighter regime might indicate greater investment in the penal subject, and help to mitigate the pains of misrecognition and neglect (Crewe & Ievins, 2021). Therefore, any attempt to model severity on the basis of these factors would have to engage with the interaction of weight with presence, and tightness with recognition, in order to figure out the relative severity added by these dimensions.

Thirdly, and more fundamentally, Crewe’s analysis does not indicate how the four dimensions of a penal regime would interact with one another to affect the severity of sentences. Should the potential mitigating effects of depth, weight, tightness and breadth be considered independently, or all at once, and how should the interaction of their affects be taken into account? This is, in fairness, a wider question in the study of aggravating and mitigating factors. We tend to treat sentencing factors as independent, atomized features of a sentence (e.g., Maslen, 2015), but of course in reality they all form part of the fabric of the sentence and the way it is likely to be experienced (e.g., Tata, 2020). So how do we properly model the likely interaction of different dimensions of punishment at sentencing, in terms of either pain units or sentence length? Again, Crewe’s discussion does not try to explore this point, and so provides no detail on it. His account is more interested in cataloging the pains of imprisonment than in measuring their typical severity, relative to one another. It is simply not part of what he has (so far) set out to do. This leaves a gap in the knowledge that his scholarship has opened up, which can be plugged with further empirical research into these questions, using a range of qualitative and quantitative methods to translate Crewe’s penology into data amenable to use in the sentencing context.

### Beyond Incommensurability: Crewe the Comparative?

A third intuitive element of Crewe’s dimensional model is that, especially with the advent of the breadth dimension (Crewe, 2015), Crewe’s analysis is amenable to discussions of pain that cut across different modes of punishment. Under the current system, we have seen that it can be difficult to *commensurate* different modes of punishment: how many weeks of probation orders are worth 6 months of imprisonment, for instance? Calculations based solely on length are inevitably unsatisfying as a way of comparing the relative severity of different modes of punishment. Prison, probation, fines, and other forms of penalty are just too qualitatively different for there to be a clear answer to the question of how much of one sentence is “worth” how much of another (e.g., Kahan, 1998). Indeed, formally, English law denies the possibility of substituting probation for prison (say): a prison sentence can only be imposed if a community order, by itself or with a fine, would be insufficient to recognize the seriousness of the offense (Criminal Justice Act 2003, s. 152(2)). In practice, as discussed above, this means that it can be difficult for judges to make the transition across the “custody threshold,” and mitigate a custodial sentence into a community one (Padfield, 2011).

Crewe’s account offers a means of reducing the problems associated with incommensurability. By focusing on the qualities of a punishment beyond its duration, his dimensions enable us to think much more broadly when comparing different modes of punishment. If we can recognize the relative depth, weight, tightness, and breadth of a given sentence, then that makes it easier to commensurate one sentence length with another. This is most obviously the case, again, if we introduce the dimensions of punishment at the stage of setting the starting range for punishment in terms of “pain units.” If we determine, for instance, that incarceration in a particular prison is worth a total of, say, 15 pain units, whereas the imposition of a community order involving a given number of requirements would be worth a total of, say, 10 pain units. Multiplying this “base number” by the length of sentence would provide a means of comparison—in our example, the community order is worth two-thirds as many pain units as imprisonment, so an equivalent sentence of imprisonment would need to be two-thirds the length of the community order.

However, the inadequacy of such an approach should be obvious. For one thing, psychological literature suggests that the longer one is subjected to unpleasant conditions such as punishment, the less painful those conditions become as one adapts to the new normality—a process known as “hedonic adaptation” (see Bronsteen et al., 2009). Conversely, there may be particular pains of long-term imprisonment (especially related to aging in prisons: Khechumyan, 2018; compare O’Donnell’s (2014, pp. 201–221) “pain quotient”), or of experiencing a continuous string of short-term sentences that leave little time as a free individual within society (Schinkel, 2014a, 2021). So, it does not follow that, to achieve equivalent penal impact, we can simply multiply the other four dimensions by the length of punishment. Indeed, further, we need to remember that the pains of punishment are extremely dynamic and react to social contexts, in terms of how that sentence is imposed (Sexton, 2015) and in terms of how it interacts with ongoing social processes and relationships in the subject’s life (Hayes, 2018). Again, Crewe’s analysis does not tell us how to work through these sorts of questions in calculating sentence length. As noted above, however, this is a common challenge facing subjectivist approaches to punishment, and it is a problem that can be solved by further empirical research. While it remains the case that Crewe’s account, by itself, does not help us to avoid the need for that further research, the dimensions do provide a means by which to structure that research agenda.

There is, however, another reason to be skeptical about Crewe’s account’s usefulness in solving the commensurability problem. This is simply because the dimensions he has described have so far existed solely within the confines of the prison, and have been designed primarily to explore the impact of differences between prison regimes in terms of the overall pain that they tend to engender. We know that there are pains of probation (e.g., Durnescu, 2011), but it does not follow that the pains imposed by non-custodial sentences are bound by the same dimensions, in the same ways and to the same extent that they do in a custodial context. To be sure, we might expect some dimensions to be relevant across multiple modes of punishment. For instance, in England and Wales, the subordination of judgments about risks and rehabilitation to psychiatric professionals has happened both within and beyond the prison estate, suggesting that the “tightness” of punishment, as a consequence of omnipresent psychological assessment, is a common experience (e.g., Canton & Dominey, 2018, pp. 114–132). It should also be remembered that with the advent of Crewe’s interest in depth, his model has incorporated the experience of release from prison on license (Crewe, 2015), an experience with many similarities to the daily experience of (at least some) subjects serving community penalties. However, it does not follow that we can understand, much less subsume, the individual pains of non-custodial punishment into a conceptual system designed to describe prisons and intimately concerned with the realities of running a prison regime.

The problem here is that non-custodial penalties have a long history of being spoken of in terms of prison. These “alternatives to prison” have tended to struggle to replace prison sentences, precisely because they set up prison as the baseline punishment, to be replaced by a (necessarily inferior or at least less “natural” supplement: Mair, 1998, p. 263; Tata, 2020, pp. 161–167). If we conceptualize the pains of punishment only in terms of patterns that have appeared in the pains of imprisonment, then we risk missing or distorting key details to make them fit into our existing model. This is part of a wider tendency amongst penologists to ignore probation (Robinson, 2016), and indeed, to ignore other non-custodial penalties like fines, which are even less well-studied. This is not to say that Crewe’s four dimensions cannot apply at all outside prison walls, but it does mean that one ought to be cautious of over-generalizing, particularly in a way that reifies prison as the “normal” penalty. Again, further research is needed; but given that that is the case, why should the conceptual starting point of that research be limited to contexts derived from 21st-Century English prisons?

## Conclusions: Ben Crewe on the Bench?

Crewe’s model offers a tantalizing glimpse of a world in which sentencing can be more responsive to the predicted experiences of the sentence imposed by its subject. We have seen, however, that significant gaps remain in Crewe’s account from the perspective of what sentencing researchers need. This is, in the strictest sense, a limitation of Crewe’s dimensions—they are limited in terms of what they can tell a sentencing judge. But then, Crewe is not writing for a sentencing context. He is a sociologist of punishment, engaging in descriptive sociology. His research does not seek to set out parameters for sentencing authorities to follow, and so we cannot reasonably criticize him if his research fails to do so. But it should give us pause in straightforwardly applying the dimensional framework to the sentencing process. The limitations of Crewe’s framework can also enlighten us as to what *is* needed if we are to achieve greater subjectivity in sentencing.

Specifically, to sum up, Crewe’s account lacks sufficient information for the sentencing context about: the quantum of the depth, weight, tightness and breadth of different punishments (that is, to be able to say not just *that* Sentence A is deeper than Sentence B, but also *how much*); the relationship between these four criteria and the length of the sentence (in terms of how much each dimension contributes to the overall severity of the sentence, and taking account of the evidence that a “tighter” or “heavier” sentence is not always more painful than a “looser” or “lighter” one); and the extent to which the pains of non-custodial penalties can be mapped onto the same dimensions, at all, or to the same extent, that Crewe has studied in relation to the pains of imprisonment.

This is, it must be said, a good problem for academics to have, because it presents an agenda for further research, rather than suggesting that existing research leads us down a blind alley. If we cannot straightforwardly take Crewe out of the prison and transplant him into the courtroom, then we have the tools to make an adaptation of his framework. To be sure, the task would be onerous, and research-intensive. Any subjectivist agenda must be, because that is the cost of trying to do justice in the complex sociological reality of punishment in human societies. But the fact that the task is difficult does not mean that it is impossible. The objectivist objection that started this enquiry, in other words, is unsustainable. The choice between penal objectivism and subjectivism is ultimately between doing difficult, intensive empirical research into the knowable subjective differences in the experience of punishment, which can inform and be synthesized into sentencing decision-making, and a system that continues in wilful ignorance of them. The more committed we are to undergirding sentencing decision-making with in-depth information about the experiences of punishment, the better-equipped we are to avoid substantive inequality and disproportionality in those impacts.

However, there is an important caveat to this strong endorsement of the practicality of penal subjectivism. As I note above, I use Crewe’s dimensions as an existing model to demonstrate how we *might* apply the insights of research into subjective experiences of punishment in a sentencing context. However, it may be that Crewe’s account obscures as much as it reveals in the sentencing context. On the one hand, Crewe’s account helps to avoid the difficulties with recognizing the plurality of pains, and clearly delineates between a pain *of punishment* and a pain *of going about one’s life* in a way that a more inductive approach to the pains of punishment would struggle with (see particularly Hayes, 2018). On the other hand, reducing the complexity and interactions of the pains of punishment to a predetermined list of dimensions risks missing or distorting key details in the experience of punishment that are likely to affect its ultimate subjective severity: when all you have is a hammer, all your problems start to look like nails (compare Hayes, 2016, pp. 734–736). The solution to this dilemma, this choice between irreducible complexity and the clarity needed for consistent sentencing decision-making, depends upon the level of arbitrariness that one is comfortable with in a sentencing regime. No matter how closely we map the pains of punishment, they will still be the pains of punishments that *have been experienced* by particular research participants, and not the pains that *will be experienced* by the current subject of sentencing. Prediction will never be perfect, and so some arbitrariness must remain. It seems to me that the level of arbitrariness ought to be reduced as much as possible, and therefore that sentencing researchers should not be limited by the framework provided by Crewe’s dimensions, useful as they are. Rather, a framework for interpreting the likely pains of punishment ought to be derived inductively from empirical data, and developed specifically so as to be comprehensible and easy to use by sentencers who may not be academic sociologists. Crewe’s research is a signpost, not a short-cut, in other words.

Importantly, the limitations of Crewe’s research for our purposes illustrates the need for deeper *interdisciplinarity* in thinking about sentencing and punishment. We will never bring penal subjectivities into the courtroom unless penal philosophers, sentencing theorists, and sociologists of punishment engage with one another. Sociologists of punishment must consider the implications of their research for sentencing, as the process by which punishment is imposed. They must be prepared to consider how subjective experiences might be compared against one another, and the extent to which they interact to affect the overall impact of the sentence on the penal subject. Greater effort must be taken to attempt to *quantify*, or otherwise compare, the qualitatively rich but subjectively unique pains of punishment. We must consider the interactions of different pains, and consider the dimensions that influence the experience of non-custodial penalties. Likewise, we must identify when the dimensions of a punishment are likely to increase and decrease the painfulness of that punishment, and under what circumstances.

Sentencing theorists and penal philosophers, meanwhile, must engage with punishment as it is experienced, rather than as they might wish it to be, recognizing that subjective differences can have significant impacts upon sentencing outcomes, individual wellbeing, and the fairness and justice of the system. We ought to engage more with the inherent variability of human experiences, and how this impacts upon the fairness of penal procedures. There is also room to consider how different aspects of a penalty’s impact can be reflected in the guidance given to judges, for instance by moderating guidelines, producing expanded lists of aggravating and mitigating factors, and clarifying the meanings of concepts like proportionality, equality and justice in a more subjective (or intersubjective) context.

Rumors of the impossibility of penal subjectivism in the courtroom have been greatly exaggerated, but to make it a practical possibility, we need to ask radically different questions, and escape our traditional disciplinary comfort zones. The question is no longer, “Can it be done?*”* but, “*Should it*?” and “If so, how?” Crewe’s work contributes to an answer to these questions—but it cannot be straightforwardly used to provide a satisfactory answer to either. By asking these questions, penal researchers of all stripes can contribute to making sentencing more responsive to subjective difference, and therefore fairer and more just in terms of the impacts of the penal State.

## Notes

1. Presently in English sentencing law, one is sentenced to imprisonment, and not to a particular prison. I do not see a way to materially incorporate subjective experience into sentencing without recognizing the differences between institutions. This would involve a great deal of practical reform. However, since I am not setting out a program for reform of one jurisdiction so much as blazing a trail for future work to do so, I will skim over this point for now, as well as other pragmatic challenges to proportionate sentencing, such as taking account of the offender’s dangerousness.
